# Impact of preoperative anemia on short- and long-term outcomes of sphincter-preserving rectal cancer surgery

**DOI:** 10.1007/s12672-025-03532-w

**Published:** 2025-09-03

**Authors:** Jung Wook Suh, Min Hyun Kim, Duck-Woo Kim, Min Hyeong Jo, In Jun Yang, Jeehye Lee, Heung-Kwon Oh, Sung-Bum Kang

**Affiliations:** 1 Department of Surgery, Dankook University Hospital, Cheonan, Korea; 2 Department of Surgery, Asan Medical Center, University of Ulsan College of Medicine, Seoul, Korea; 3 Department of Surgery, Seoul National University Bundang Hospital, 300 Gumi-dong Bundang-gu, Seongnam-si, 13620 Gyeonggi-do Korea; 4 Department of Surgery, Chungnam National University Hospital, Daejeon, Korea; 5 Department of Surgery, Yonsei University Yongin Severance Hospital, Yongin, Korea

**Keywords:** Preoperative anemia, Rectal cancer, Rectal surgery, Sphincter preservation, Complication, Outcome

## Abstract

**Background:**

Preoperative anemia is associated with an increased risk of postoperative complications and poor survival in colorectal cancer; however, its effects on long-term outcomes in sphincter-preserving rectal surgery remain unclear. Therefore, we analyzed the correlation among preoperative anemia, postoperative complications and surgical outcomes in sphincter-preserving rectal cancer surgeries.

**Methods:**

Data from patients who underwent sphincter-preserving surgery for stage I–III rectal cancer between 2011 and 2015 were reviewed. Anemia was defined as a preoperative baseline hemoglobin concentration < 12.5 g/dL in men and < 11.5 g/dL in women. Disease-free survival (DFS), overall survival (OS) and 30-day overall complications according to the Clavien–Dindo (CD) classification were compared between the anemia and non-anemia groups.

**Results:**

Overall, 120 of the 638 patients (18.8%) analyzed had preoperative anemia. The most common postoperative complications were ileus (6.7%), urinary retention (5.0%), wound complications (4.7%), and anastomotic leakage (2.7%). The anemia group exhibited significantly more overall complications, major complications, and anastomotic leaks compared to the non-anemia group. However, the 5-year DFS and OS were comparable between groups. Male sex, ileostomy, vascular invasion, and anemia correlated with overall complications.

**Conclusions:**

Preoperative anemia was linked to postoperative complications, especially anastomotic leaks, but did not affect OS or DFS. Thus, our results suggest that rectal cancer surgery requires tailored management in patients with anemia.

## Introduction

Rectal cancer often invades adjacent organs because of its anatomical location in the pelvic cavity [1–3]. Such invasion presents considerable surgical challenges, particularly an increased risk of intraoperative bleeding during pelvic dissection for advanced or recurrent tumours [4–6]. Sphincter-preserving rectal cancer surgery is associated with improved quality of life—including better sexual and urinary function—while achieving oncologic outcomes comparable to abdominoperineal resection [7–10]. However, anastomosis in sphincter preserving surgery carries inherent risks of leakage due to factors, such as male sex, obesity, tobacco habit, preoperative radiation, postoperative anemia, hypoalbuminemia, and late initiation of enteral nutrition, which are linked to higher rates of local recurrence and poorer survival [11–14].

Preoperative anemia affects approximately 40% patients with colorectal cancer (CRC) [15, 16], including approximately 20% with rectal cancer and 50% with colon cancer [15, 17, 18]. Previous studies have also demonstrated that preoperative anemia is an independent risk factor for postoperative complications, transfusions, prolonged hospital stay, and increased morbidity and mortality within 30 days in patients with CRC [19–21]. Furthermore, oncological outcomes have been linked to a decline in both overall survival (OS) and recurrence-free survival (RFS) in CRC [18, 22].

A 2019 multicenter study from the Netherlands reported a lower 3-year overall survival rate in rectal cancer patients with preoperative anemia, but it did not demonstrate a clear association with postoperative complications such as anastomotic leakage. This may have been due to the inclusion of many patients who underwent abdominoperineal resection with permanent stoma formation, and thus did not receive an anastomosis [23]. To address this limitation, we sought to investigate the relationship between preoperative anemia, postoperative complications, and oncologic outcomes, focusing exclusively on sphincter-preserving rectal cancer surgeries and excluding colon cancer cases.

## Materials and methods

### Study population and procedure

Data of patients who underwent curative resection with sphincter preservation for rectal cancer between January 2011 and December 2015 at a tertiary referral hospital were retrospectively analyzed. The rectum was defined as being 15 cm from the anal verge. Patients with histologically proven adenocarcinoma at TNM stage ≤ 3 who underwent curative resection with anastomosis, such as low anterior resection (LAR), ultralow anterior resection (uLAR), or intersphincteric resection (ISR) with or without ileostomy, were included. Patients who underwent surgery for stage IV rectal cancer, hereditary cancer, or inflammatory bowel disease and those who underwent transanal local excision, Hartmann’s operation, or abdominopelvic resection were excluded from the study. Hemoglobin levels were measured at the time of admission for surgery. Anemia was defined as the lowest quartile (bottom 25%) of hemoglobin levels in the study cohort, corresponding to values of ≤ 12.5 g/dL in men and < 11.5 g/dL in women. This study was approved by our Institutional Review Board of Seoul National University Bundang Hospital (approval number B-2412-944-103) and was conducted in accordance with the ethical standards set forth in the Declaration of Helsinki. The need for informed patient consent was waived due to the retrospective nature of this study.

All patients underwent colonoscopy, computed tomography (CT), and tumor marker evaluation. Magnetic resonance imaging (MRI) was performed to assess resectability and for preoperative TNM staging. The goal of each surgery was R0 resection, which was achieved through a total mesorectal excision in all cases, followed by resection and anastomosis to ensure a minimum distal resection margin of 1 cm. Surgery was conducted at tertiary referral hospitals by colorectal surgeons who perform > 200 CRC surgeries annually. LAR was performed in cases of upper-mid rectal cancer. uLAR was performed for low rectal cancer located within 6 cm of the anal verge, when ISR was not applicable [24]. Ileostomy was performed to guarantee the safety of the anastomosis, when required. All patients underwent postoperative follow-up in accordance with the standard surveillance protocols for rectal cancer [25]. Physical examinations and serum carcinoembryonic antigen measurements were performed every 3 months during the first 2 years, followed by every 6 months over a 5–8-year period. CT scans of the abdomen, pelvis, and chest were performed at intervals of 3–6 months during the initial 2 years and every 6–12 months thereafter. Endoscopic evaluations were conducted one year postoperatively and biennially thereafter. When recurrence was suspected, liver or pelvic MRI and positron emission tomography-CT (PET-CT) were performed as indicated.

### Data and study outcomes

Data regarding the patients’ baseline characteristics, including age, sex, body mass index (BMI), and American Society of Anesthesiologists (ASA) classification, was obtained. Additionally, endoscopic findings, such as the length of the tumor location at the anal verge, and treatment-related parameters, including the type of operation (LAR, uLAR, or ISR), stoma formation status, administration of neoadjuvant chemoradiotherapy, and application of adjuvant chemotherapy, were collected.

The primary outcome was the 5-year OS, defined as the time from the date of surgery to the date of death or the last follow-up. Secondary outcomes included the 5-year disease-free survival (DFS), recurrence, complications above Clavien–Dindo (CD) grade I [26], factors associated with complications, and factors related to survival. The 5-year DFS was measured from the date of surgery to the date of recurrence, last follow-up, or death. Recurrence was identified based on radiological evaluations, including CT, MRI, and PET, or histological confirmation via endoscopic examination. Major complications were defined as CD Grade ≥ III.

### Statistical analysis

Normally distributed continuous variables are expressed as means with standard deviations, whereas non-normally distributed variables are presented as medians with interquartile ranges. The Student’s t-test and the Mann–Whitney *U* test were used to compare normally and non-normally distributed variables, respectively. Categorical variables are presented as counts with percentages and were compared using the χ^2^ test or Fisher’s exact test, depending on their distribution. Time-dependent variables were analyzed using the Kaplan–Meier method and compared using the log-rank test. Complications were assessed by univariate and multivariate analyses using logistic regression analysis. Factors influencing survival were assessed by univariate and multivariate analyses using the Cox proportional hazards regression model. All statistical tests were two-sided, and statistical significance was defined as *P* < 0.05. All statistical analyses were performed using R (R version 4.2.1; R Core Team, Vienna, Austria).

## Results

### Patient characteristics and pathologic features

A total of 638 patients underwent sphincter preserving surgery for rectal cancer. Laparoscopic surgery was performed in 88.9% (567/638) cases, with only six cases being converted to open surgery. The median patient age was 63 years (range, 54–72 years), with a male predominance of 63.9% (*n* = 408). Neoadjuvant chemoradiotherapy was administered to 26.2% patients (167/638), while 60.8% patients (388/638) received adjuvant chemotherapy. The median follow-up period was 4.5 years, and the 5-year OS rate was 93.0%.

The anemia group was older (69 vs. 61 years; *P* < 0.001), had a lower BMI (22.2 vs. 23.5 kg/m²; *P* < 0.001), and exhibited a poorer general physical status according to the ASA classification compared to the non-anemia group. Additionally, the tumor was located further from the anus in patients in the anemia group (9.5 vs. 8.0 cm; *P* = 0.028) who had a higher T stage, although no significant difference was observed in the N stage between the groups. The TNM stage was generally higher in the anemia group, however, the rate of adjuvant chemotherapy was not significantly different between the two groups (61.1% vs. 60.7%; *P* = 1.000) (Table 1).

**Table 1:** Baseline characteristics of patients in the anemia and non-anemia groups

Variable	Anemia (*n* = 162)	Non-anemia (*n* = 476)	*P*-value
Age (years)	69 (59–75)	61 (53–70)	< 0.001
Sex, female	58 (35.8)	172 (36.1)	1.000
BMI, kg/m^2^	22.2 (20.6–24.2)	23.5 (21.7–25.6)	< 0.001
*ASA PS classification*			< 0.001
1	34 (21.0)	171 (35.9)	
2	117 (72.2)	296 (62.2)	
3	11 (6.8)	9 (1.9)	
Tumor location	9.5 (6–14)	8.0 (5–10)	0.028
*Operation*			0.368
LAR	122 (75.3)	333 (70.0)	
ULAR	28 (17.3)	93 (19.5)	
ISR	12 (7.4)	50 (10.5)	
Ileostomy	88 (54.3)	51.7 (24.6)	0.624
Neoadjuvant chemoradiotherapy	43 (26.5)	124 (26.1)	0.984
*T stage*			< 0.001
1	26 (16.0)	115 (24.2)	
2	28 (17.3)	112 (23.5)	
3	91 (56.2)	238 (50.0)	
4	17 (10.5)	11 (2.3)	
*N stage*			0.099
0	91 (56.2)	304 (63.9)	
+	71 (43.8)	172 (36.1)	
*TNM stage*			0.022
0 or 1	46 (28.4)	193 (40.5)	
2	45 (27.8)	111 (23.3)	
3	71 (43.8)	172 (36.1)	
Lymphatic invasion	41 (25.3)	102 (21.5)	0.368
Venous invasion	32 (19.8)	87 (18.3)	0.773
Perineural invasion	62 (38.3)	152 (32.0)	0.173
Adjuvant Chemotherapy	99 (61.1)	289 (60.7)	1.000

Data are presented as N (%) or median (interquartile range)

*BMI* body mass index, *ASA PS* American Society of Anesthesiologists physical status, *LAR* low anterior resection, *ULAR* ultralow anterior resection, *ISR* intersphincteric resection, *TNM* tumor–node–metastasis

### Complications

The overall complication rate was 26.6% (170/638), with a significantly higher incidence in the anemia group compared to the non-anemia group (36.4% vs. 23.3%, *P* = 0.001). Complications in the anemia group occurred frequently in the following order: ileus (6.8%, 11/162), urinary retention (6.8%, 11/162), and anastomosis-related complications (5.6%, 9/162). The most common complications in the non-anemic group were ileus (7.1%, 34/476), wound complications (6.3%, 30/476), and urinary retention (3.6%, 17/476). The anemia group also had a higher complication severity based on the CD classification. Moreover, the incidence of anastomotic leakage, a major complication, was significantly higher in the anemia group than that in the non-anemia group (5.6% vs. 1.7%; *P* = 0.019) (Table 2). Complication risk factors were also analyzed across the study population. In the univariate analysis, male sex, anemia, type of operation, ileostomy formation, neoadjuvant chemoradiotherapy, and vascular invasion were significantly associated with complications (Table 3). Whereas, male sex (odds ratio [OR] 1.58, 95% confidence interval [CI]: 1.05–2.37, *P* = 0.027), anemia (OR 1.88, 95% CI 1.26–2.80, *P* = 0.002), ileostomy formation (OR 1.87, 95% CI 1.28–2.73, *P* = 0.001), and vascular invasion (HR 1.57, 95% CI 1.01–2.45, *P* = 0.044) were identified as significant risk factors for complications in the multivariate analysis (Table 3). The subgroup analysis indicates that patients without anemia but with an ileostomy have an OR of 2.77 (95% CI, 1.76–4.37; *P* < 0.001), signifying a substantial adverse effect on complications. Furthermore, patients with anemia exhibited ORs of 3.43 (95% CI, 1.88–6.25; *P* < 0.001) without an ileostomy and 3.41 (95% CI, 1.93–6.03; *P* < 0.001) with an ileostomy, both indicating significant negative impacts on complications.

**Table 2:** Complications observed in the anemia and non-anemia groups

	Anemia (*n* = 162)	Non-anemia (*n* = 476)	*P*-value
*Clavien–Dindo classification*			0.011
1	25 (15.4)	51 (10.7)	
2	24 (14.8)	(9.5)	
≥ 3	10 (6.2)	15 (3.2)	
*Anastomotic complication*	9 (5.6)	8 (1.7)	0.019
1	1	1	
2	3	0	
≥ 3	5	7	
*Overall complications*	59 (36.4)	111 (23.3)	0.001
Ileus	11 (6.8)	34 (7.1)	
Urinary retention	11 (6.8)	17 (3.6)	
Wound complications	8 (4.9)	30 (6.3)	
Anastomotic complications	9 (5.6)	8 (1.7)	
Chylous ascites	5 (3.1)	8 (1.5)	
Pulmonary complications	4 (2.5)	4 (0.8)	
Phlebitis	3 (1.9)	2 (0.4)	
Cardiac complications	4 (2.5)	0	
Luminal bleeding	1 (0.6)	3 (0.6)	
Fever of unknown origin	2 (1.2)	1 (0.2)	
Bowel ischemia	0	1 (0.2)	
Urinary tract infection	0	2 (0.4)	
Burn injuries	0	1 (0.2)	
Renal failure	1 (0.6)	1 (0.2)	

Data are presented as N (%)

**Table 3:** Univariate and multivariate logistic regression analysis for risk factors affecting complications

	Univariate analysis		Multivariate analysis	
	OR (95% CI)	*P*-value	OR (95% CI)	*P*-value
*Age (years)*				
≥ 70	1.23 (0.85–1.78)	0.279		
*Sex*				
Male	1.89 (1.28–2.79)	0.001	1.58 (1.05–2.37)	0.027
BMI (kg/m^2^)				
≥ 25	0.69 (0.46–1.04)	0.075		
*ASA PS classification*				
1	Ref.			
2	1.01 (0.69–1.48)	0.942		
3	0.92 (0.32–2.65)	0.877		
Anemia	1.88 (1.28–2.77)	0.001	1.88 (1.26–2.80)	0.002
*Tumor location*				
≥ 5	Ref.			
< 5	1.39 (0.89–2.16)	0.148		
*Operation*				
LAR	Ref.			
ULAR	1.10 (0.70–1.74)	0.679		
ISR	2.21 (1.28–3.83)	0.005		
Ileostomy	2.00 (1.39–2.87)	< 0.001	1.87 (1.28–2.73)	0.001
Neoadjuvant chemoradiotherapy	1.52 (1.03–2.23)	0.033		
*TNM stage*				
1	Ref.			
2	1.16 (0.74–1.82)	0.526		
3	1.00 (0.67–1.50)	0.997		
Lymphatic invasion	1.06 (0.70–1.62)	0.779		
Vascular invasion	1.54 (1.00–2.38)	0.048	1.57 (1.01–2.45)	0.044
Perineural invasion	1.31 (0.91–1.90)	0.152		

*BMI* body mass index, *ASA PS* American Society of Anesthesiologists physical status, *LAR* low anterior resection, *ULAR* ultralow anterior resection, *ISR* intersphincteric resection, *HR* hazard ratio, *CI* confidence interval, *TNM* tumor–node–metastasis, *Ref*. reference

### Oncologic outcomes

**Fig. 1:**
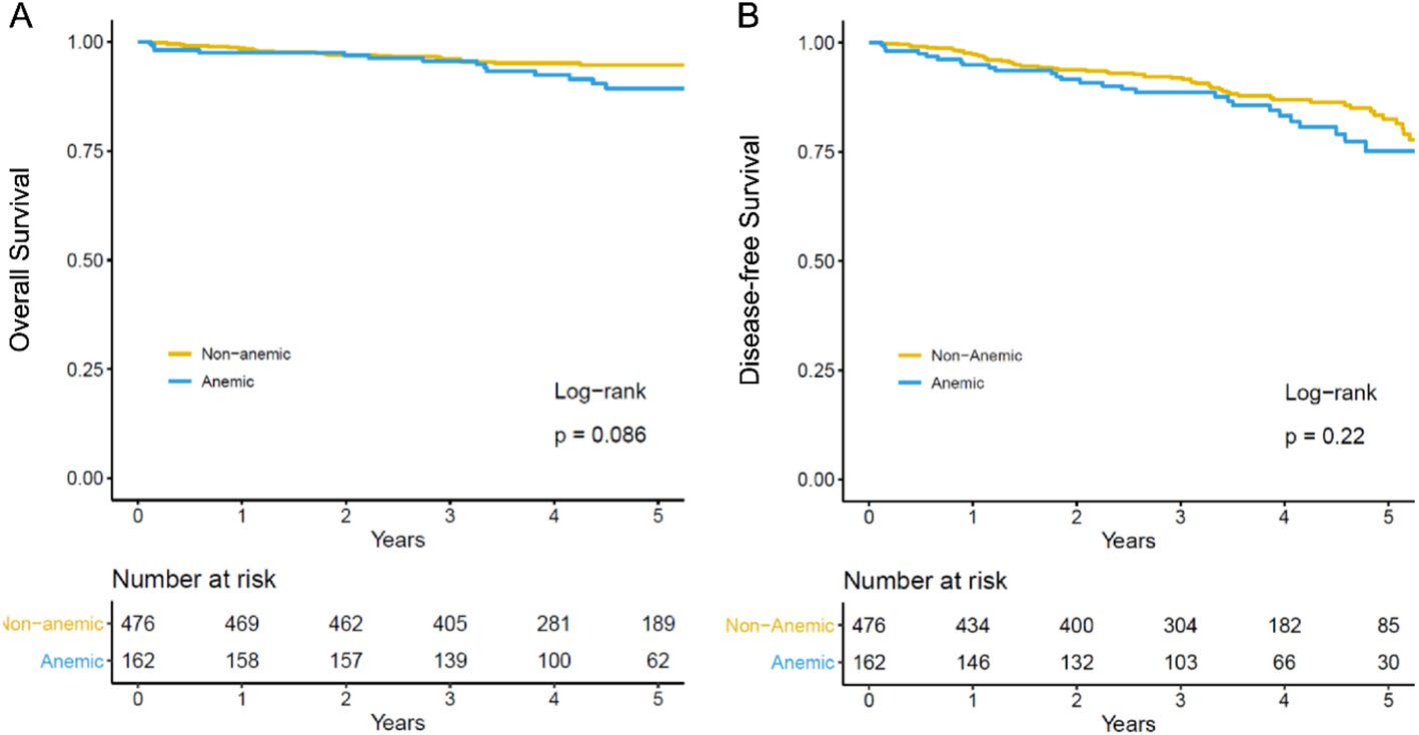
Kaplan–Meier curve of the overall survival and disease-free survival associated with preoperative anemia after sphincter preservation rectal surgery (**a**) overall survival (**b**) disease-free survival

**Fig. 2:**
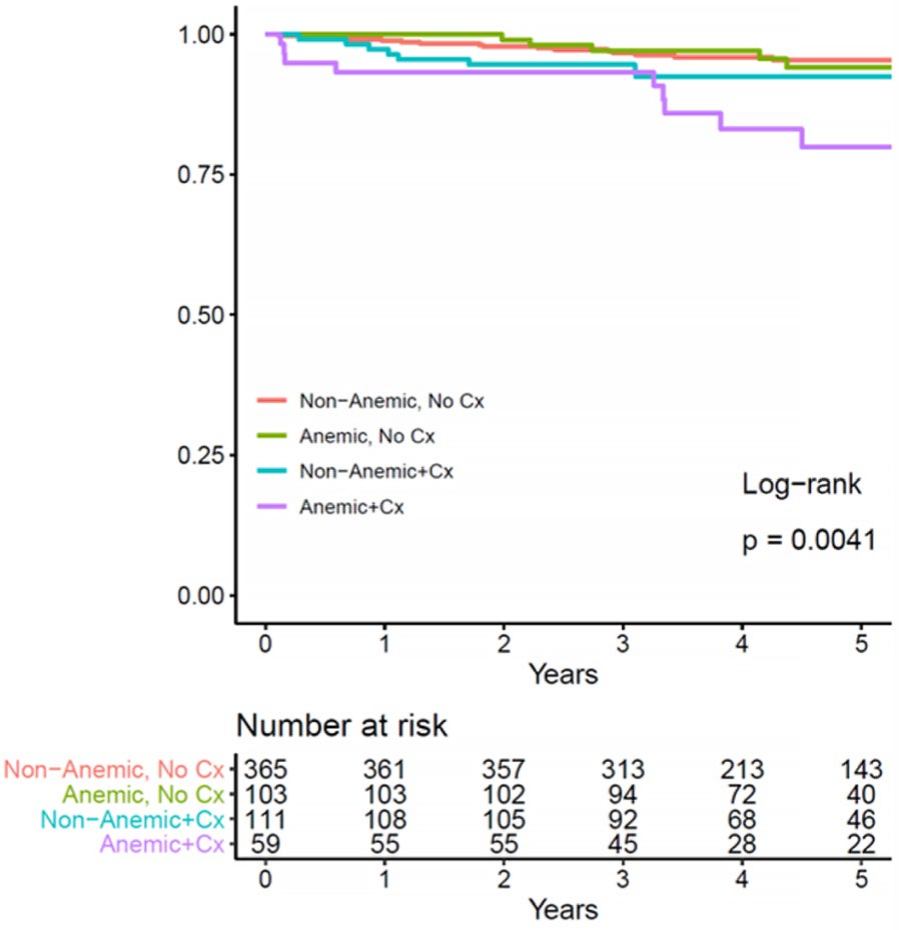
Kaplan–Meier curve of the overall survival by subgroup based on the presence of preoperative anemia and postoperative complications after sphincter preservation rectal surgery

No significant differences were observed in the 5-year OS rates between the anemic and non-anemic groups (5-year OS: 91.5% vs. 89.3%, *P* = 0.086) (Fig. 1A). Similarly, the 5-year DFS rates did not differ significantly between the anemic and non-anemic groups (5-year DFS: 86.3% vs. 82.5%, *P* = 0.216) (Fig. 1B). Factors affecting OS in the univariate analyses included age, ASA classification, complications, TNM stage, lymphatic invasion, vascular invasion, and perineural invasion (Table 4). Age (hazard ratio [HR] 4.45, 95% CI 2.03–9.74, *P* = 0.001), ASA PS classification (HR 6.43, 95% CI 1.47–28.0, *P* = 0.013), TNM stage (HR 2.97, 95% CI 1.14–7.78, *P* = 0.027), and vascular invasion (HR 3.18, 95% CI 1.50–6.74, *P* = 0.003) were identified as significant risk factors for complications affecting OS and adjuvant chemotherapy completion in the multivariate analyses (HR 0.33, 95% CI 0.15–0.71, *P* = 0.004) (Table 4). In addition, anemia with complications was associated with significantly poorer survival outcomes compared with the absence of anemia without complications in the OS subgroup analysis. Specifically, patients with anemia and complications had an HR of 4.02 (95% CI 1.76–9.19, *P* = 0.001), signifying a substantial adverse effect on survival. However, patients with anemia without complications (HR 1.12, 95% CI 0.41–3.09, *P* = 0.823) and those with non-anemia with complications (HR 1.77, 95% CI 0.75–4.17, *P* = 0.194) did not demonstrate significant associations with survival (Fig. 2).

**Table 4:** Univariate and multivariate Cox proportional hazards analysis for risk factors affecting overall survival

	Univariate analysis		Multivariate analysis	
	HR (95% CI)	*P*-value	HR (95% CI)	*P*-value
*Age (years)*				
≥ 70	6.25 (3.02–12.9)	< 0.001	4.45 (2.03–9.74)	< 0.001
*Sex*				
Male	1.33 (0.92–1.94)	0.134	1.97 (0.92–4.23)	0.082
*BMI (kg/m*^2^)				
≥ 25	1.07 (0.53–2.17)	0.852		
*ASA PS classification*				
1	Ref.		Ref.	
2	3.76 (1.32–10.7)	0.013	2.24 (0.74–6.65)	0.150
3	11.2 (2.81–44.9)	0.001	6.43 (1.47–28.0)	0.013
Anemia	1.77 (0.91–3.45)	0.090		
Complications	2.44 (1.28–4.67)	0.007	1.92 (0.99–3.75)	0.055
*Tumor location*				
≥ 5	Ref.			
< 5	1.32 (0.60–2.89)	0.484		
*Operation*				
LAR	Ref.			
ULAR	1.57 (0.73–3.38)	0.252		
ISR	1.17 (0.40–3.36)	0.778		
Neoadjuvant chemoradiotherapy	0.77 (0.35–1.67)	0.516		
*TNM stage*				
1	Ref.		Ref.	
2	1.01 (0.33–3.08)	0.992	0.86 (0.26–2.81)	0.799
3	3.11 (1.40–6.92)	0.006	2.97 (1.14–7.78)	0.027
Lymphatic invasion	3.00 (1.57–5.74)	0.001	1.87 (0.92–3.85)	0.085
Vascular invasion	3.32 (1.73–6.37)	< 0.001	3.18 (1.50–6.74)	0.003
Perineural invasion	1.91 (1.00–3.64)	0.050		
Adjuvant chemotherapy	0.59 (0.31–1.13)	0.113	0.33 (0.15–0.71)	0.004

*BMI* body mass index, *ASA PS* American Society of Anesthesiologists physical status, *LAR* low anterior resection, *ULAR* ultralow anterior resection, *ISR* intersphincteric resection, *HR* hazard ratio, *CI* confidence interval, *TNM* tumor–node–metastasis, *Ref* reference

## Discussion

In this study, we analyzed the relationship among preoperative anemia, complications, and long-term outcomes in patients undergoing rectal cancer surgery with sphincter preservation. Preoperative anemia was identified as a factor influencing complications, with a significantly higher incidence of anastomotic leakage in patients with anemia than in those without. Moreover, although preoperative anemia did not directly affect OS or DFS, an increase in postoperative complications due to anemia is thought to ultimately affect the long-term outcomes.

Preoperative anemia was identified as an independent risk factor for postoperative complications and prolonged hospital stays in a large multicenter study of patients undergoing surgery for CRC [19]. Conversely, another study conducted on patients with CRC found that preoperative anemia independently predicted the need for postoperative blood transfusions but showed no association with 30-day postoperative complications. Additionally, postoperative blood transfusion emerged as an independent risk factor for 30-day postoperative complications [22]. In contrast, a Dutch study involving patients who underwent rectal surgery, including those who underwent sacrifice of the anus or procedures without anastomosis, reported that preoperative anemia was not independently associated with postoperative complications of rectal cancer [23]. However, our study of patients undergoing rectal surgery with preservation of the anus and anastomosis, found that preoperative anemia was associated with a 1.88-fold increase in the risk of postoperative complications. Moreover, a separate analysis of anastomotic leakage further confirmed this significant association. In a prospective multicenter study of patients undergoing colorectal surgery, preoperative anemia was identified as the single most important contributor to anastomotic leakage (OR 5.4), with the underlying mechanism attributed to impaired tissue oxygen delivery (tissue hypoxia) [27]. Currently, ongoing studies are evaluating whether active correction of preoperative anemia and subsequent increases in hemoglobin levels are actually associated with a reduction in postoperative complications, and the results are eagerly anticipated. In this context, anastomotic leakage can result in permanent stoma formation during rectal cancer surgery. Therefore, it is advisable to consider proactive anastomotic protection strategies, such as the creation of a temporary ileostomy, in patients with rectal cancer with preoperative anemia.

Temporary ileostomy is often recommended to ensure the stability of the anastomosis [28]; however, it is associated with additional risks, such as wound and parastomal complications [29]. Accordingly, the risk of complications further increased in patients with preoperative anemia who underwent ileostomy formation in our study. While factors such as male sex and vascular invasion are non-modifiable, preoperative anemia is a modifiable risk factor that requires proactive assessment and correction prior to surgery to mitigate these risks. Our findings highlight the necessity of tailored management strategies in patients with preoperative anemia undergoing sphincter-preserving rectal cancer surgery. In particular, more active consideration of temporary ileostomy may help reduce the risk of major complications such as anastomotic leakage.

Previous meta-analyses reported that preoperative anemia is significantly associated with a reduction in both OS and RFS in patients with CRC [18]. Furthermore, a large-scale study which included patients with colon and rectal cancer identified preoperative anemia as an independent risk factor for decreased OS and RFS. Additionally, studies focusing exclusively on patients with rectal cancer showed that preoperative anemia, with an HR of 1.4, was independently associated with a poorer 3-year OS [22]. Thus, although the clinical relevance of anemia as an isolated factor may be limited, it can be considered an indicator of overall frailty in patients with rectal cancer [23] However, this study found that preoperative anemia did not significantly affect OS or DFS; therefore, it was not analyzed as an influencing factor in patients undergoing sphincter-preservation rectal surgery. Moreover, although the risk of OS increased by 1.92 times for postoperative complications, this increase was not statistically significant. Nevertheless, the subgroup analysis revealed that patients with preoperative anemia who experienced postoperative complications had a four-fold increased risk in terms of OS compared to those without either condition. These findings underscore the importance of meticulous management of patients with anemia who develop postoperative complications.

The standard of care for locally advanced rectal cancer has shifted from single radical resection to the current multimodality treatment, including standard chemoradiotherapy and total neoadjuvant therapy. Previous systematic reviews have established neoadjuvant chemotherapy as a safe and tolerable option for locally advanced CRC, highlighting oncologic benefits such as tumor downstaging and high R0 resection rates [30–32]. Additionally, a randomized controlled trial comparing total neoadjuvant therapy, which administers chemotherapy prior to neoadjuvant chemoradiotherapy, with standard chemoradiotherapy demonstrated improved outcomes with total neoadjuvant therapy, including a pathologic complete response rate of 28% versus 12% and a 3-year disease-free survival rate of 76% versus 69% [33]. Moreover, a meta-analysis of RCTs showed that the combination of short-course radiotherapy and consolidation chemotherapy had a relative risk of 1.07 (95% CI, 1.01–1.14) for an improved 3-year OS. However, at 5 years, no treatment demonstrated a statistically significant advantage over other treatments in terms of OS [34]. The NICHE study evaluated the efficacy of preoperative immunotherapy in patients with locally advanced microsatellite instability-high (MSI-H)/deficient Mismatch Repair (MMR) CRC, who were administered a combination of nivolumab and ipilimumab before surgery, resulting in major pathological responses in 95% patients and a pathological complete response in 60% patients, indicating that neoadjuvant immunotherapy can elicit significant treatment responses in this patient population [35]. Whereas, in our study, standard chemoradiotherapy was administered in approximately 20% of the cases; however, our retrospective study has limitations, as it was conducted prior to the aforementioned research. At that time, our center opted for aggressive surgical interventions for upper rectal cancer, or when resectability was deemed feasible. Since then, total neoadjuvant therapy has become increasingly standardized in subsequent studies; therefore, it is imperative to investigate the impact of pre-treatment anemia, as well as anemia that develops or persists post-treatment, on complications and oncologic outcomes.

In this study, adjuvant chemotherapy was confirmed to be an important independent protective factor associated with OS [25, 36–39]. At our center, the need for neoadjuvant chemoradiotherapy is determined through preoperative evaluation, while postoperative adjuvant chemotherapy is administered based on clinical staging. Therefore, adjuvant chemotherapy was administered according to the pathological staging in cases in which neoadjuvant chemotherapy was not administered, particularly in patients with stage II high-risk features or those with stage III disease. Moreover, worse survival rates were reconfirmed when appropriate chemotherapy could be administered due to factors such as patient refusal, underlying conditions such as liver cirrhosis, or poor postoperative performance. Consequently, proactive recommendations and the facilitation of chemotherapy are essential to ensure optimal outcomes, unless unavoidable patient conditions impede chemotherapy.

Furthermore, pelvic dimensions differ between men and women. A previous study reported significant differences in sacral breadth, transverse diameter of the pelvic inlet, while the distance between femoral heads is greater in women than that in men [40]. These anatomical differences contribute to the increased complexity of performing rectal cancer surgeries in men compared with women. Additionally, previous studies focusing exclusively on patients with rectal cancer reported that the female sex is associated with lower rates of postoperative complications and mortality [23]. Similarly, our study found that, although male sex was not identified as a factor influencing OS, it was independently associated with a 1.58-fold increase in postoperative complications.

Nonetheless, this study has some limitations. First, the retrospective nature of the study introduced an inherent selection bias that could not be entirely avoided. Therefore, future studies should employ prospective designs or statistical adjustments to address this issue. Second, recent modifications in rectal cancer therapy merit further attention. Only 26.2% patients in our study received preoperative chemoradiotherapy, a proportion significantly lower than the 38.1% observed in the pathological staging. Given the downstaging effects of neoadjuvant chemoradiotherapy, the actual number of patients who underwent this treatment is likely underestimated. Furthermore, this discrepancy is likely to increase as total neoadjuvant therapies emerge and expand their coverage. Finally, in this study, anemia was defined as the lowest quartile of hemoglobin levels within the cohort, which differs from the definitions used in previous studies. This underscores the need for a precise and standardized definition of clinically relevant preoperative anemia. Moreover, stratification by anemia grade may provide valuable insights; however, this was beyond the scope of the present analysis and remains a subject for future research.

## Conclusions

In our study, we did not observe a clear association between preoperative anemia and long-term survival in patients undergoing sphincter-preserving rectal cancer surgery. However, anemia seemed to increase the risk of short-term complications, particularly anastomotic leakage. We believe that careful perioperative management in anemic patients is important and should be viewed as part of a broader effort to improve long-term outcomes.

## Data Availability

All data generated in the course of this study will be made available upon specific request to the corresponding author.
